# Genome-Wide Marker–Trait Analysis of Agronomic and Biomass-Related Traits in an Interspecific Miscanthus F_1_ Population Grown on Reclaimed Mine Land

**DOI:** 10.3390/plants15203172

**Published:** 2026-10-16

**Authors:** Liang Xiao, Xuhong Zhao, Cong Lin, Hui Feng, Zili Yi, Hongmei Huang, Mingxi Liu

**Affiliations:** 1 College of Bioscience and Biotechnology, Hunan Agricultural University, Changsha 410128, China; xiaoliang@hunau.edu.cn (L.X.);; 2 Shandong Key Laboratory of Peanut Breeding, Shandong Peanut Research Institute, Qingdao 266100, China; 3 Botanical Garden, Institute of Botany, The Chinese Academy of Sciences, Beijing 100093, China; 4 China National Botanical Garden, Beijing 100093, China; 5 College of Biological Engineering, Binzhou Polytechnic University, Binzhou 256603, China; 6 College of Agronomy, Hunan Agricultural University, Changsha 410128, China

**Keywords:** *Miscanthus sacchariflorus*, *Miscanthus lutarioriparius*, interspecific hybrid, biomass, plant architecture, marker–trait association, reclaimed mine land, marker × year interaction

## Abstract

Miscanthus is a perennial bioenergy crop suited to marginal and degraded land, yet relatively little is known about the genetic basis of biomass-related variation in interspecific hybrids across years. We evaluated 100 F_1_ genotypes from *Miscanthus sacchariflorus × M. lutarioriparius* for two growing seasons at a reclaimed mine site and analyzed 16 agronomic, biomass-related, and physiological traits using an existing linkage map of 461 SNP marker positions across 19 linkage groups. No single-trait peak exceeded its trait-specific 5% permutation threshold. The clearest repeated suggestive signal occurred in the proximal region of LG9: marker2342 (11.571 cM) showed the strongest across-year signals for plant height and tiller length, while the adjacent marker2343 (10.561 cM) gave the strongest across-year signal for tiller number. Four correlated architecture traits were also summarized by principal component analysis. PC1 explained 73.2–76.6% of their variation, and in 2019 marker0580 on LG3 (12.516 cM) exceeded the genome-wide threshold (LOD 3.67 vs. 3.47). This association remained significant after removal of the single rare genotype-class observation but was not reproduced in 2018; formal marker × year interaction tests confirmed temporal heterogeneity for all four component traits. Biomass and physiological signals were weaker and less repeatable. Thus, the study identifies a repeated but moderate LG9 pattern and a stronger, temporally variable LG3 signal expressed at this reclaimed site; it does not establish loci specifically conferring adaptation to reclaimed mine land. These regions provide priorities for validation using larger populations, denser marker coverage, additional years, and contrasting environments before their value for marker-assisted breeding can be assessed.

## 1. Introduction

Miscanthus is a perennial C_4_ grass valued for high biomass production and relatively low input requirements, making it a candidate biomass crop for marginal land [1,2,3,4,5]. Reclaimed mine land is one setting in which this feature is relevant, as conventional crop production is often constrained, while field studies have shown that Miscanthus can establish and produce biomass under such conditions [6,7,8,9]. East Asia is also a major center of Miscanthus diversity. *M. sacchariflorus* contributes broad adaptation, whereas *M. lutarioriparius* is notable for vigorous growth and high biomass potential. Genomic and population-transcriptomic resources are now available for *M. lutarioriparius* [10,11,12], making crosses between these species useful for examining segregating variation in growth and biomass-related traits.

Biomass yield is determined by several correlated traits, including plant height, tiller number, stem dimensions, leaf development, phenology, and individual tiller biomass [13,14,15,16,17]. Mapping studies in *M. sinensis* and *M. sacchariflorus* have identified loci associated with many of these components, but effects are not always repeatable among years or environments [14,15,16]. For perennial field traits, repeatability across seasons can be as informative as the size of a single-year effect. Multi-year data are therefore important when genomic signals for growth and biomass are interpreted.

SNP-based linkage maps have enabled studies of quantitative variation in highly heterozygous *Miscanthus* populations. RNA-seq-derived markers have been used to build maps covering 19 linkage groups, and F_1_ populations have been used to map plant height, flowering, leaf traits, and biomass productivity [15,18,19,20]. Much less is known about *M. sacchariflorus* × *M. lutarioriparius* populations, especially when they are grown under semiarid reclaimed-mine conditions. The same interspecific cross used here has previously been evaluated for growth, biomass production, and physiological performance on reclaimed mine land [8], and was also examined by transcriptomic analysis in this field environment [9]. However, its mapped genetic variation has not been evaluated systematically against the agronomic and physiological phenotypes measured over multiple years.

Here we evaluated 100 F_1_ genotypes from *M. sacchariflorus* × *M. lutarioriparius* over two growing seasons at a reclaimed mine site on the Loess Plateau of China and used an existing 461-marker linkage map as the positional framework for marker–trait scans. We asked which genomic signals were repeatedly observed across years and correlated traits and which were temporally variable. The analyses identified a repeated but sub-threshold plant-architecture signal in proximal LG9 and a stronger 2019-specific multivariate architecture signal at marker0580 on LG3. Because all genotypes were evaluated at a single reclaimed site, these associations are interpreted as genetic effects expressed in this environment rather than as loci specifically conferring adaptation to reclaimed mine land.

## 2. Materials and Methods

### 2.1. Plant Materials and Field Experiment

An interspecific F_1_ mapping population was developed from a cross between *M. sacchariflorus* (female parent, accession 02487, collected from Gansu Province, China) and *M. lutarioriparius* (male parent, accession 04081, collected from Changsha, Hunan Province, China). The cross was made in 2010, and hybrid authenticity was previously confirmed using diagnostic SSR markers [21]. The original field experiment comprised 113 F_1_ genotypes together with the two parents, as described previously [8]. The present genetic analysis was restricted to the 100 F_1_ genotypes represented in the retained linkage-map genotype dataset and having corresponding field phenotypic records. The archived records do not permit reliable retrospective determination of why the remaining 13 field-evaluated genotypes were not included in the linkage-map dataset.

The field experiment was established in 2018 on a flat reclaimed area at the Xiaoyi aluminium mine in Shanxi Province, China (37.12° N, 111.45° E), on the Loess Plateau [8]. The site consisted of reclaimed soil overlying mine waste or gravel and was nutrient-poor; before planting, mean soil organic carbon and total nitrogen in the upper soil layer were 3.61 and 0.33 g kg^−1^, respectively [8]. The original experiment described the field as having homogeneously reclaimed soil. In May 2018, 10 clonal rhizomes (7–10 cm) per F_1_ genotype and 15 rhizomes per parent were assigned to planting positions according to a completely randomized layout within an approximately 60 × 20 m field, with 1 m × 1 m spacing [8]. Rhizomes were watered at planting, after which no supplemental irrigation was applied, and no fertilizer was used during the experiment [8]. Annual precipitation was 388 mm in 2018, including 172 mm in August, and 443 mm in 2019, including 209 mm during August and September. From October 2018 to April 2019, daily minimum ground temperature was below 0 °C for 148 days and below −15 °C for 19 days from December 2018 to February 2019; daily maximum air temperature was below 10 °C for 105 days from November 2018 to March 2019 [8]. No formal pre-plant spatial survey of soil depth, compaction, fertility, or water availability was conducted, and the archived dataset does not contain fine-scale spatial covariates for retrospective spatial correction.

### 2.2. Phenotypic Evaluation

Phenotypic measurements were obtained during the 2018 and 2019 growing seasons. Sixteen agronomic, biomass-related, and physiological traits were evaluated: plant height, tiller length, tiller diameter, tiller number, leaf angle, leaf length, leaf width, leaf area, single-tiller fresh weight, whole-plant fresh weight, photosynthetic rate, stomatal conductance, intercellular CO_2_ concentration, transpiration rate, instantaneous water-use efficiency (WUEe; A/E), and intrinsic water-use efficiency (WUEi; A/g_s_). For each genotype, three surviving plants were randomly selected and followed across the two growing seasons [8]. These individual plants served as biological replicates for field phenotyping; their measurements were averaged arithmetically to obtain one genotype-level value for each trait in each year, which was the analytical unit used in the marker–trait analyses. Plant height was measured from the soil surface to the youngest leaf with a ligule on the tallest stem, tiller diameter was measured 3–5 cm above ground, and tiller number was counted within 1 m^2^. Biomass-related traits were measured in late November of each growing season. Marked plants were harvested leaving approximately 3–5 cm of stubble, and all tillers within a 1-m^2^ area centered on the planting position were collected. Harvested material was placed in labelled paper bags and returned to the laboratory for immediate determination of fresh weight. Whole-plant fresh weight was the total fresh weight of all harvested tillers within the 1-m^2^ area; single-tiller fresh weight represented the fresh weight of an individual tiller. 

Leaf gas-exchange traits were measured using a LI-6400XT portable photosynthesis system (LI-COR, Lincoln, NE, USA) equipped with a 2 × 3 cm natural-light leaf chamber. Measurements were made on the fourth fully expanded leaf between 10:00 and 12:00 h. Ambient temperature and photon flux density were allowed to track field conditions, while the reference CO_2_ concentration was maintained at 400 μmol mol^−1^. Measurements were recorded after gas-exchange parameters had stabilized, and three instrument readings were logged for each plant. Measurements were conducted on 1–6 July, 18–27 August, and 22–28 September 2018, and on 23–25 July, 17–18 August, and 21–24 September 2019 [8].

### 2.3. SNP Genotyping and Linkage-Map Framework

The present study used an existing genetic linkage map previously developed for this interspecific F_1_ population as the positional framework for the marker–trait analyses. The retained dataset contains 461 mapped SNP marker positions. The archived individual-level genotype matrix permits the observed genotype-class configurations to be summarized directly: 160 markers showed AA/AB classes, 124 showed AB/BB classes, 173 showed all three AA/AB/BB classes, and four were invariant as AB in the retained 100-genotype dataset. These labels describe observed downstream genotype classes and are not treated as formal phase-specific CP segregation codes. The original pre-filtering marker dataset, marker-specific parental phase/CP segregation-code records, and marker-to-transcript correspondence are no longer available; therefore, the exact numbers historically removed at individual filtering steps cannot be reconstructed reliably, and candidate genes were not assigned retrospectively.

The existing linkage map contains 461 mapped SNP positions across 19 linkage groups (LGs), consistent with the 19 chromosomes in the haploid complement of Miscanthus (2*n* = 38). Genetic distances in the existing map were expressed in centimorgans using the Kosambi mapping function. The map spans 3215.56 cM; LG lengths range from 95.03 to 231.95 cM, with 12–48 markers per LG and mean adjacent-marker intervals of 4.54–15.61 cM. Because the parents are highly heterozygous and the progeny form a full-sib F_1_ family, marker segregation was interpreted under a cross-pollinator (CP) framework appropriate for outcrossing species [22]. In the present analyses, observed genotype classes at each marker were treated as categorical levels without assuming a common F_2_-type AA/AB/BB segregation pattern or imposing an additive genetic model.

### 2.4. Statistical Analysis

For each genotype and year, the three plant-level measurements were first averaged arithmetically to obtain a genotype-level annual phenotype. For traits measured in both 2018 and 2019, across-year genotype estimates were obtained from a fixed-effects model containing genotype and year, with genotype treated as fixed. For every genotype entering a given two-year analysis, observations were available as complete 2018–2019 pairs; consequently, the across-year least-squares estimate was numerically identical to the arithmetic mean of the two annual genotype-level values. Four genotypes lacked observations in both years for single-tiller fresh weight and whole-plant fresh weight and were excluded from those analyses (N = 96); all other two-year traits had complete records for 100 genotypes. Leaf angle was measured only in 2019 (N = 100). For continuity with the analytical workflow, the across-year least-squares means are referred to as BLUE values. The across-year analysis served as the combined multi-year analysis; the two annual observations from the same genotype were not treated as independent samples.

Descriptive statistics included sample size, mean, standard deviation, coefficient of variation, skewness, and Pearson kurtosis. Because the fixed-effects across-year estimates were numerically identical to the two-year arithmetic genotype means for all genotypes with data, the same across-year genotype values were used for descriptive summaries, phenotypic distributions, and the primary across-year marker–trait analyses. Pearson correlations were used to assess relationships among traits and cross-year phenotypic consistency. Missing observations were not imputed. Year-specific descriptive statistics and cross-year correlations are provided in Supplementary Table S2.

Genome-wide marker–trait scans were conducted separately for the 2018, 2019, and BLUE datasets using the mapped marker set. At each marker, the observed genotype classes retained in the analysis dataset were fitted as a categorical factor without imposing a common F_2_-type AA/AB/BB segregation model or an additive linear relationship. This categorical treatment is consistent with the full-sib F_1_ origin of the population, in which different markers may represent different segregation configurations under the CP framework. Marker support was summarized as a logarithm-of-odds (LOD) score from the residual sums of squares of the marker and intercept-only models, and phenotypic variance explained (PVE) was the R^2^ of the corresponding single-marker model. Genome-wide significance was determined by 1000 phenotype permutations; the 95th percentile of the maximum genome-wide LOD distribution was used as the 5% threshold [23]. Peaks below this threshold were treated as suggestive associations.

Detection power was evaluated by simulation using the retained genotype matrix. Of the 461 mapped marker positions, four had no genotype variation in the retained analysis dataset and were therefore non-informative for this simulation, leaving 457 polymorphic markers. Ten thousand null phenotypes were simulated to estimate the 5% genome-wide maximum-LOD threshold, followed by 5000 simulations for each assumed marker effect (PVE = 5–30%). In each simulation, a polymorphic marker with complete genotypes was selected as the simulated causal marker, an additive effect was scaled to the specified PVE, normally distributed residual variation was added, and the phenotype was scanned using the same categorical marker model. Power was the proportion of simulations in which the simulated causal marker exceeded the genome-wide threshold (Figure S3). Because this design assumes a directly genotyped causal marker, it represents an optimistic scenario; detection power would be lower when an assayed marker is incompletely linked to the underlying causal locus.

For genomic regions recurring across related traits or datasets, selected markers were examined by observed genotype-class comparisons. Genotype-class frequencies were inspected before post hoc interpretation. Pairwise differences for adequately represented classes were tested with Welch’s *t*-tests, standardized effects were expressed as Cohen’s d, and multiple related tests were adjusted using the Benjamini–Hochberg false discovery rate (FDR). Classes represented by fewer than five individuals were not used for quantitative genotype-effect interpretation; such rare classes were either excluded in a predefined robustness analysis, as for marker0580, or the corresponding marker was not interpreted further at the genotype-class level. Neighboring markers in the proximal LG9 interval (approximately 4.2–16.8 cM) were examined as local marker comparisons. Because parental phase assignments are unavailable, no phased haplotype analysis was attempted.

Principal component analysis (PCA) was used to summarize correlated plant-architecture traits (plant height, tiller length, tiller diameter, and tiller number) separately for the 2018, 2019, and BLUE datasets. Traits were standardized before PCA, and PC1 was oriented so that the plant-height loading was positive. PC1 was then analyzed as a composite phenotype in genome-wide scans of all 461 markers, with significance assessed by 1000 phenotype permutations [24,25]. Exact PC1 loadings and variance explained are reported in Supplementary Table S5.

For the strongest 2019 PC1 association, rare-class robustness was assessed by excluding, marker by marker, observed genotype classes represented by fewer than five individuals and repeating both the genome-wide scan and 1000-permutation procedure. At marker0580, the retained downstream genotype coding comprised classes labelled AA (n = 1), AB (n = 19), and BB (n = 80); these labels are used here only to identify the observed classes in the retained analysis dataset and are not intended to imply a common F_2_ segregation model for the mapping population. Exclusion of the single rare-class individual retained 99 individuals for reanalysis.

Temporal heterogeneity was tested formally using repeated-measures generalized estimating equations (Gaussian family, exchangeable working correlation), with F_1_ genotype as the clustering unit and fixed effects for marker genotype class, year, and marker × year interaction. For marker0580, the single AA-labelled genotype was excluded from the interaction test because a one-individual class cannot provide a stable estimate, leaving AB (n = 19) and BB (n = 80). The model was fitted separately to plant height, tiller length, tiller diameter, and tiller number, and the four interaction *p* values were adjusted using the Benjamini–Hochberg FDR procedure. The same four-trait interaction analysis was performed for marker2342 (AA = 33, AB = 67).

## 3. Results

### 3.1. Phenotypic Variation and Trait Distribution

A total of 16 agronomic, biomass-related, and physiological traits were evaluated in the interspecific Miscanthus F_1_ population. Annual phenotypes were genotype-level arithmetic means of three measured plants. Plant height, tiller length, tiller diameter, tiller number, leaf length, leaf width, leaf area, and the physiological traits had complete annual observations for all 100 mapped F_1_ genotypes. Single-tiller fresh weight and whole-plant fresh weight were available for 96 genotypes in both years because four genotypes lacked both biomass measurements, while leaf angle was measured only in 2019 (N = 100). The population exhibited broad continuous variation across the measured traits (Table 1).

Biomass-related traits showed considerable variation among the F_1_ individuals. Whole-plant fresh weight exhibited a broad phenotypic range, with some progeny values extending beyond the parental point estimates. Because parental variation was not formally incorporated into a test of transgressive segregation, these observations are described as broad phenotypic segregation rather than as evidence of transgressive inheritance. Single-tiller fresh weight likewise showed continuous quantitative variation across the F_1_ population; this distribution is consistent with quantitative variation but does not identify the parental origin or direction of allelic effects.

Plant architecture traits, including plant height, tiller length, tiller diameter, tiller number, leaf length, leaf width, and leaf area, also displayed continuous distributions across the F_1_ population. Plant height and tiller-related traits showed particularly broad quantitative variation, providing phenotypic variation for the subsequent marker–trait analyses.

Physiological traits associated with photosynthetic performance and water-use efficiency, including photosynthetic rate, stomatal conductance, intercellular CO_2_ concentration, transpiration rate, instantaneous water-use efficiency (WUEe), and intrinsic water-use efficiency (WUEi), also varied widely among F_1_ individuals, extending the phenotypic range available for marker–trait analyses.

Cross-year correlations showed moderate phenotypic stability for the four principal plant-architecture traits: plant height (r = 0.491), tiller length (r = 0.527), tiller diameter (r = 0.485), and tiller number (r = 0.684) were all positively correlated between 2018 and 2019 (all *p* < 0.001). In contrast, year-to-year correlations were weak for photosynthetic rate (r = 0.171, *p* = 0.090), WUEe (r = 0.014, *p* = 0.892), and WUEi (r = 0.048, *p* = 0.638), indicating greater temporal plasticity in these physiological measurements.

**Figure 1:**
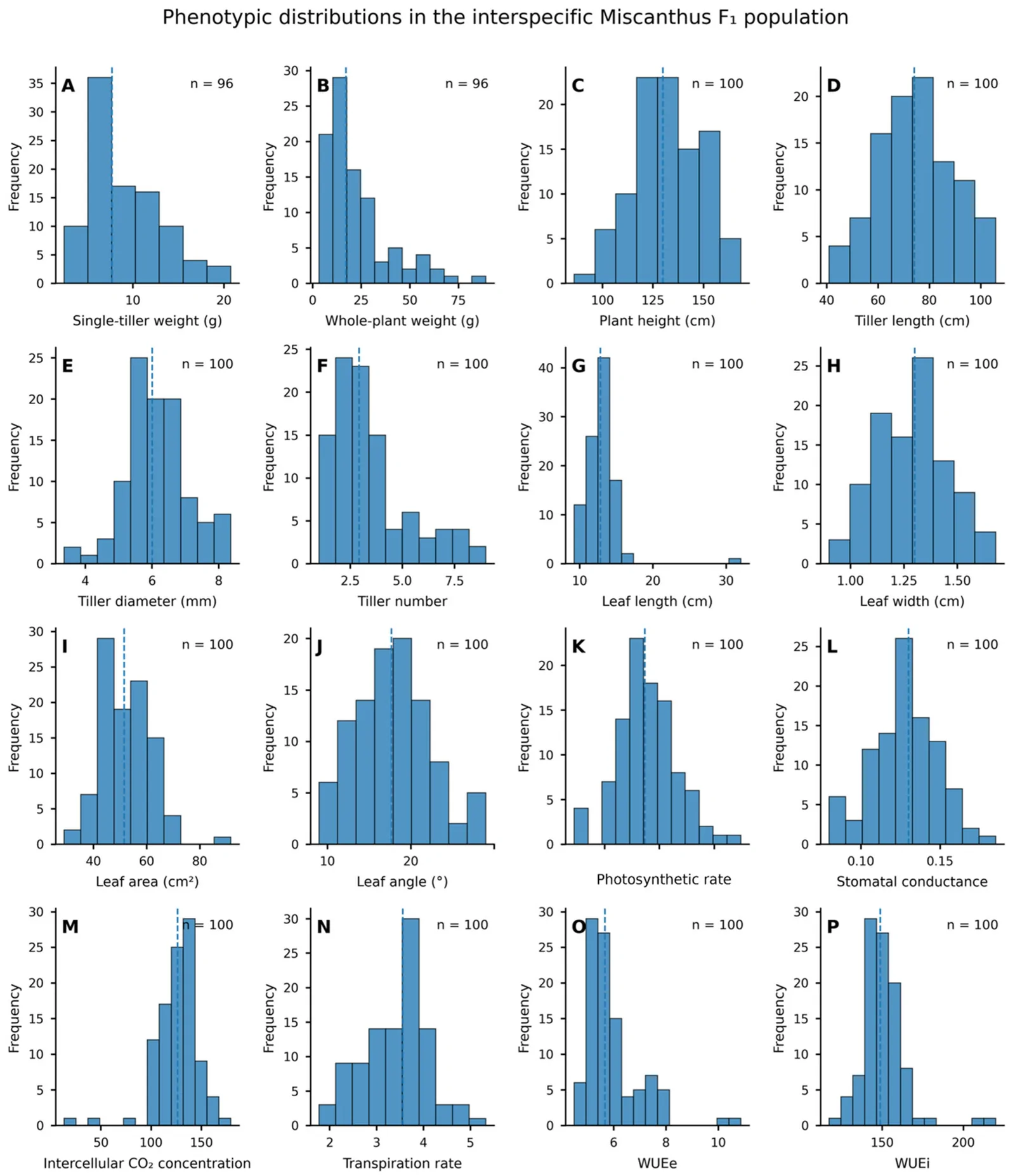
Phenotypic distributions of 16 agronomic, biomass-related, and physiological traits in the interspecific Miscanthus F_1_ population. Distributions are shown for the interspecific F_1_ population derived from *M. sacchariflorus × M. lutarioriparius*; trait-specific sample sizes are indicated in each panel. For traits measured in both 2018 and 2019, values represent the mean phenotype of each individual across the two growing seasons; leaf angle was measured in 2019 only. Dashed vertical lines indicate the median of each trait. Panels represent (**A**) single-tiller fresh weight, (**B**) whole-plant fresh weight, (**C**) plant height, (**D**) tiller length, (**E**) tiller diameter, (**F**) tiller number, (**G**) leaf length, (**H**) leaf width, (**I**) leaf area, (**J**) leaf angle, (**K**) photosynthetic rate, (**L**) stomatal conductance, (**M**) intercellular CO_2_ concentration, (**N**) transpiration rate, (**O**) instantaneous water-use efficiency (WUEe), and (**P**) intrinsic water-use efficiency (WUEi).

Most traits showed continuous phenotypic variation, consistent with quantitative inheritance, although several biomass-related, morphological, and physiological traits deviated significantly from normality (Figure 1). Rank-based inverse normal transformation was applied to selected traits to assess the robustness of the major marker–trait associations (Figure S1).

### 3.2. Genetic Linkage Map Used for Genome-Wide Marker–Trait Analysis

The genetic linkage map used as the positional framework for genome-wide marker–trait analysis comprised 461 mapped SNP positions assigned to 19 linkage groups (LGs), consistent with the 19 chromosomes in the haploid complement of Miscanthus (2*n* = 38). The present study did not reconstruct the linkage map; it used the retained marker positions from the previously constructed map. Marker identities and genetic positions are listed in Supplementary Table S1.

Because the mapping population was derived from two highly heterozygous Miscanthus parents, marker segregation is interpreted under the CP (cross-pollinator) framework. In the retained 100-genotype matrix, 160 markers showed AA/AB classes, 124 showed AB/BB classes, 173 showed all three AA/AB/BB classes, and four were invariant as AB. These are observed genotype-class configurations in the archived analysis dataset rather than reconstructed formal CP segregation codes; the original parental phase assignments are not available.

**Figure 2:**
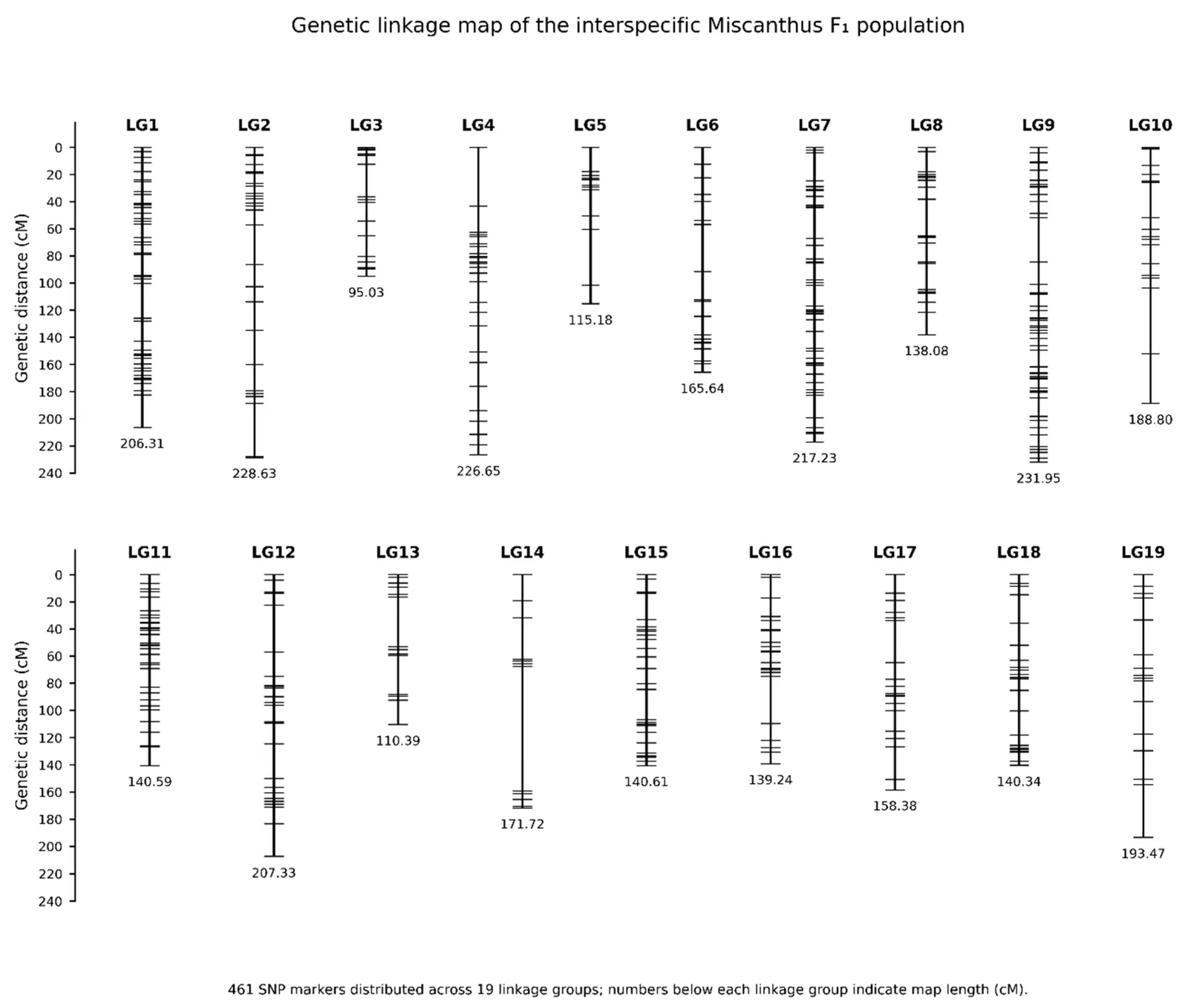
Genetic linkage map of the interspecific Miscanthus F_1_ population. LG1–LG19 denote linkage groups 1–19, corresponding to the 19 chromosomes in the haploid complement of Miscanthus (2*n* = 38). The map comprised 461 mapped SNP positions. Marker positions are shown in centimorgans (cM). The total map length was 3215.56 cM, with linkage-group lengths ranging from 95.03 cM (LG3) to 231.95 cM (LG9). The map was used as the positional framework for subsequent genome-wide marker–trait association analyses.

The total genetic length of the linkage map was 3215.56 cM, with individual linkage groups ranging from 95.03 cM (LG3) to 231.95 cM (LG9). The number of markers per linkage group varied from 12 (LG5 and LG14) to 48 (LG9), with an average of 24.26 markers per LG. The mean interval between adjacent markers ranged from 4.54 cM (LG11) to 15.61 cM (LG14) (Table 2), indicating variation in marker density across linkage groups (Figure 2).

Marker coverage was not uniform across the 19 linkage groups. LG5, LG14, and LG19 contained maximum adjacent-marker gaps of 41.05 cM, 91.63 cM, and 38.83 cM, respectively, whereas LG9 contained 48 markers with a mean interval of 4.94 cM. Because the original upstream marker-filtering and segregation records are not available, the causes of these large intervals cannot be determined from the retained map and are not inferred here. The uneven marker density limits mapping resolution and the ability to distinguish closely linked effects.

### 3.3. Genome-Wide Marker–Trait Association Analysis

**Figure 3:**
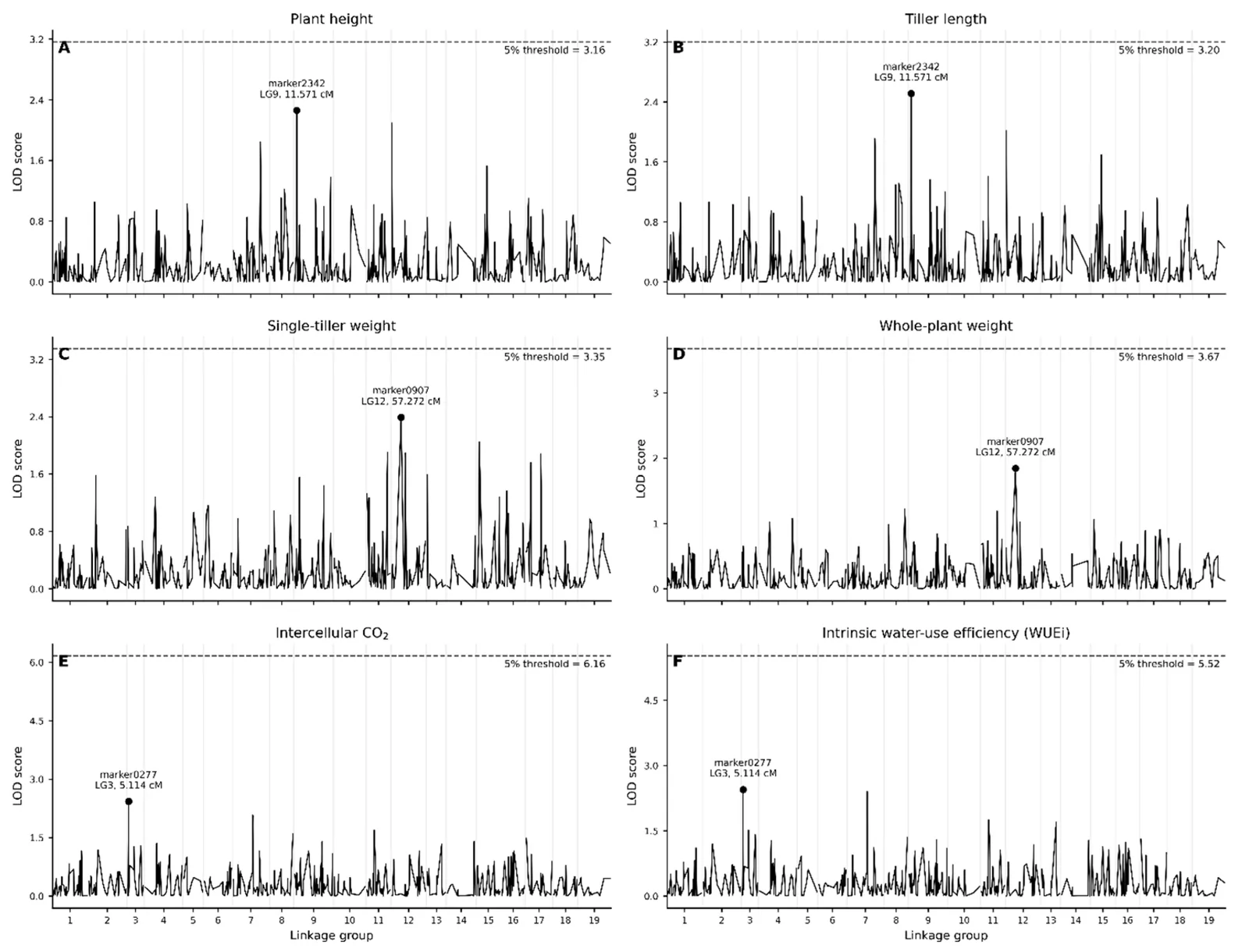
Genome-wide marker–trait association profiles for representative agronomic, biomass-related, and physiological traits in the interspecific Miscanthus F_1_ population. Genome-wide scans were conducted using BLUE values and 461 SNP markers distributed across 19 linkage groups. Panels show LOD profiles for (**A**) plant height (cm), (**B**) tiller length (cm), (**C**) single-tiller fresh weight (g), (**D**) whole-plant fresh weight (g), (**E**) intercellular CO_2_ concentration (μmol mol^−1^), and (**F**) intrinsic water-use efficiency (WUEi; μmol CO_2_ mol^−1^ H_2_O). Horizontal dashed lines indicate the trait-specific 5% genome-wide significance thresholds determined by 1000 permutations. Trait units identify the phenotypes represented in each panel; the plotted vertical axis is the unitless LOD score and the horizontal axis is genetic position. Although none of the individual-trait peaks exceeded the corresponding genome-wide threshold, repeated suggestive signals occurred on LG9 for plant architecture traits, LG12 for biomass-related traits, and LG3 for physiological traits.

Genome-wide marker–trait scans were conducted for the 16 agronomic, biomass-related, and physiological traits using the retained mapped marker set across 19 linkage groups. Across-year adjusted genotype means (BLUE values) were used as the primary phenotypes for traits measured in both years, and year-specific scans were additionally examined to assess temporal consistency. Trait-specific 5% genome-wide significance thresholds were determined using 1000 phenotype permutations. None of the single-trait association peaks exceeded the corresponding permutation threshold; thus, no genome-wide significant single-trait marker association was detected under the present population size and marker density (Figure 3). The leading peaks from the 2018, 2019, and across-year scans are summarized in Supplementary Table S3.

Despite the absence of genome-wide significant single-trait associations, several suggestive signals were shared by biologically related traits (Table 3). The clearest pattern for plant architecture occurred in the proximal region of LG9, where marker2342 at 11.571 cM showed the strongest BLUE-based associations with plant height (LOD = 2.26; PVE = 9.9%) and tiller length (LOD = 2.51; PVE = 10.9%), while the adjacent marker2343 at 10.561 cM represented the strongest signal for tiller number (LOD = 2.13; PVE = 9.4%). For biomass-related traits, marker0907 on LG12 at 57.272 cM was the strongest BLUE-based marker for both single-tiller fresh weight and whole-plant fresh weight, explaining approximately 10.8% and 8.5% of phenotypic variation, respectively. For physiological traits, marker0277 on LG3 at 5.114 cM represented the strongest BLUE-based association for both intercellular CO_2_ concentration and intrinsic water-use efficiency (WUEi), with PVE values of 10.6% and 10.7%, respectively. A further suggestive association for transpiration rate was detected at marker3163 on LG9.

Because none of these single-trait peaks reached the trait-specific genome-wide threshold, they were treated as suggestive marker–trait associations rather than significant QTLs. The proximal LG9 region showed recurring signals across correlated plant-architecture traits and was therefore examined further through genotype-class comparisons; this recurrence is not treated as independent replication because the component traits are correlated.

### 3.4. Genotype Effects Within the Repeated Suggestive Plant-Architecture Cluster on LG9

**Figure 4:**
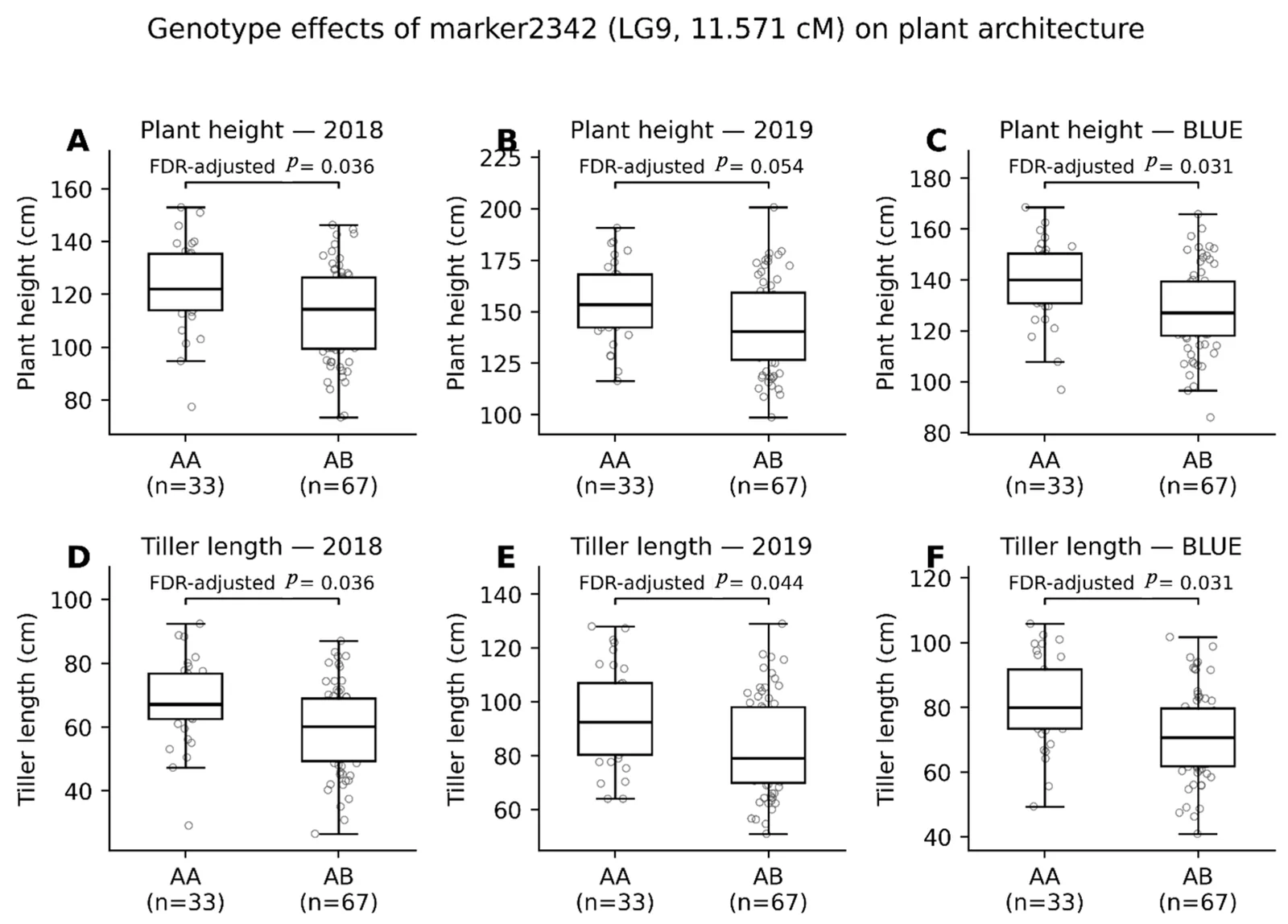
Genotype effects of marker2342 on plant height and tiller length in the interspecific Miscanthus F_1_ population. Marker2342 is located at 11.571 cM on LG9. Genotypic differences are shown for plant height in (**A**) 2018, (**B**) 2019, and (**C**) BLUE values, and for tiller length in (**D**) 2018, (**E**) 2019, and (**F**) BLUE values. Boxes indicate the interquartile range with the median shown by the central line, and individual points represent F_1_ genotype-level phenotypic means. Sample sizes are indicated below each genotype class. *p*-values were adjusted for multiple comparisons using the Benjamini–Hochberg false discovery rate (FDR) procedure.

Within the proximal LG9 cluster identified above, marker2342 at 11.571 cM showed the strongest BLUE-based associations with plant height and tiller length, whereas the adjacent marker2343 at 10.561 cM marked the strongest BLUE-based signal for tiller number. We therefore examined phenotypic differences among marker2342 genotype classes separately for 2018, 2019, and the across-year BLUE values (Figure 4).

Individuals in the AA class at marker2342 were generally taller and had longer tillers than those in the AB class. In 2018, mean plant height was approximately 123.5 cm for AA individuals compared with 112.6 cm for AB individuals, a difference of approximately 10.9 cm. A similar difference was observed in 2019 (154.8 vs. 143.0 cm), resulting in an across-year BLUE difference of approximately 11.3 cm. Tiller length showed the same directional pattern: AA individuals had tillers approximately 8.6 cm longer than AB individuals in 2018 and 11.4 cm longer in 2019, with an across-year BLUE difference of approximately 10.0 cm. After FDR adjustment, genotype-class differences remained significant for plant height in the 2018 and BLUE datasets and for tiller length in the 2018, 2019, and BLUE datasets (Figure 4). Formal repeated-measures marker × year tests were non-significant for plant height (*p* = 0.825) and tiller length (*p* = 0.417), the two traits for which marker2342 showed the principal repeated suggestive signals. The interaction was also non-significant for tiller diameter (*p* = 0.788) but was significant for tiller number (*p* = 0.00567; FDR-adjusted *p* = 0.0227). Thus, marker2342 should not be described as uniformly stable across all architecture traits. The neighboring marker2343 captured the strongest BLUE-based tiller-number signal within the same LG9 interval, but its observed classes were highly unbalanced (AA = 1, AB = 99), so genotype-class effects at marker2343 were not interpreted further. The proximal LG9 region is therefore interpreted as a repeated but moderate suggestive association region, with the strongest evidence for cross-year consistency involving plant height and tiller length rather than plant architecture as a whole.

### 3.5. Multivariate Analysis Identifies a Significant but Year-Dependent Plant-Architecture Association on LG3

Because plant height, tiller length, tiller diameter, and tiller number were strongly correlated and jointly contributed to overall plant architecture, we performed principal component analysis (PCA) to integrate these traits into a composite measure of vegetative growth. The four traits were standardized prior to PCA, and analyses were conducted separately using the 2018, 2019, and BLUE datasets. PC1 explained 73.20%, 74.17%, and 76.57% of the total variation in 2018, 2019, and the BLUE dataset, respectively. The corresponding PC1 loadings for plant height, tiller length, tiller diameter, and tiller number were 0.557, 0.576, 0.507, and 0.318 in 2018; 0.540, 0.562, 0.537, and 0.322 in 2019; and 0.539, 0.558, 0.518, and 0.360 for BLUE. All four traits therefore contributed in the same direction, with the first three traits contributing most strongly, indicating that higher PC1 scores represented generally taller plants with longer and thicker tillers together with greater tiller production (Supplementary Table S5).

**Figure 5:**
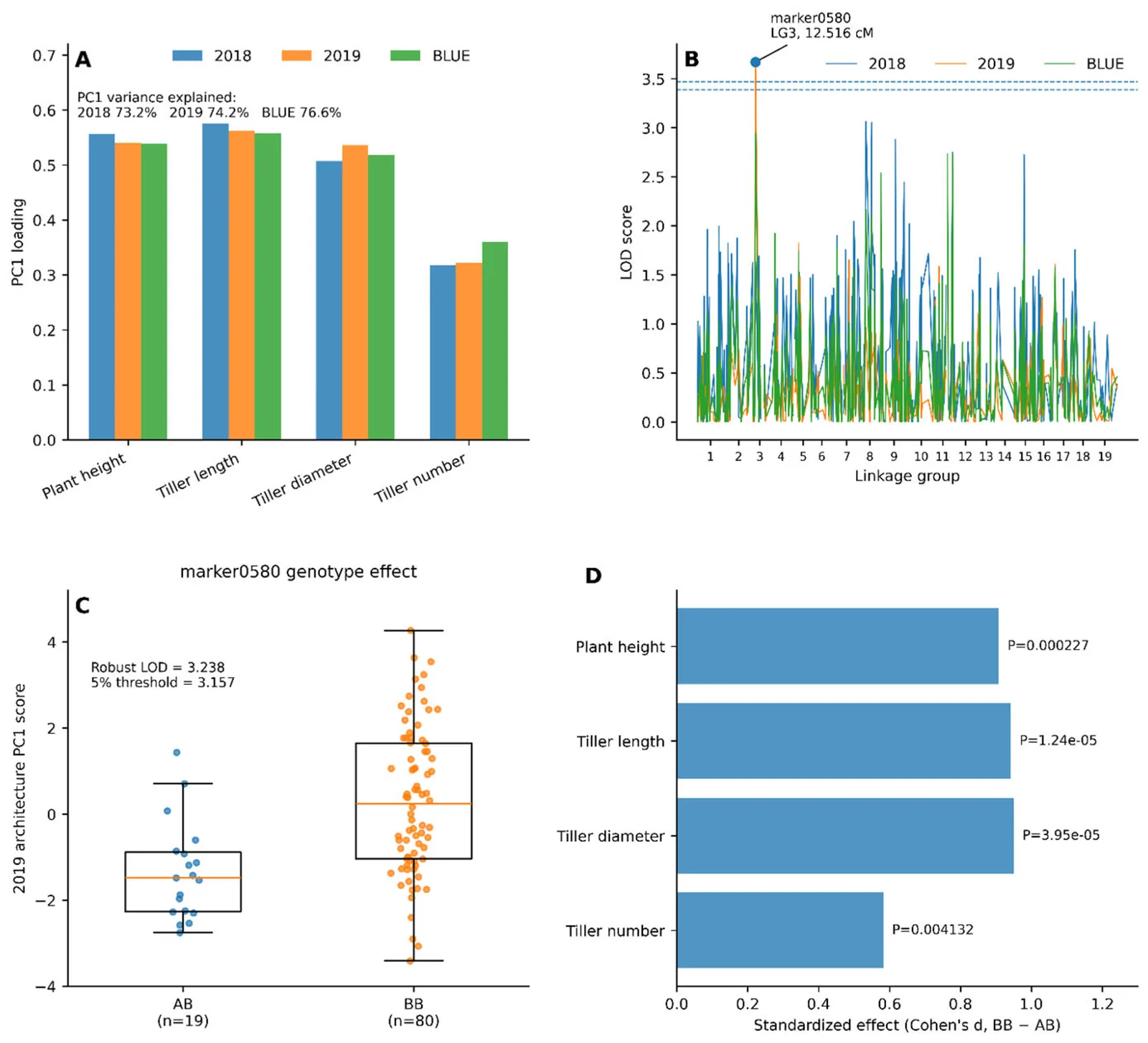
Multivariate analysis of plant architecture traits and the genome-wide significant association at marker0580 on LG3. (**A**) Principal component analysis (PCA) of four plant architecture traits, including plant height (cm), tiller length (cm), tiller diameter (mm), and tiller number (count per m^2^). Bars show the PC1 loadings of each trait for the 2018, 2019, and BLUE datasets. PC1 explained 73.20%, 74.17%, and 76.57% of the total variation in 2018, 2019, and the BLUE dataset, respectively. All four traits showed positive loadings on PC1. (**B**) Genome-wide marker-association profiles for architecture PC1 based on 461 SNP markers distributed across 19 linkage groups. The strongest association in 2019 was detected at marker0580 on LG3 (12.516 cM), with a LOD score of 3.67, exceeding the 5% genome-wide significance threshold of 3.47 determined by 1000 permutations. The corresponding peak was weaker in the BLUE dataset (LOD = 2.95) and was not detected as the major peak in 2018. The two horizontal dashed lines indicated the 5% genome-wide significance thresholds: LOD = 3.47 for both 2018 and 2019 (upper line) and LOD = 3.39 for BLUE (lower line). (**C**) Distribution of 2019 architecture PC1 scores between the AB and BB genotype classes at marker0580. The rare AA genotype (n = 1) was excluded from the robustness analysis. After exclusion, marker0580 remained significant, with a LOD score of 3.24, exceeding the corresponding 5% permutation threshold of 3.16. Points represent F_1_ genotype-level phenotypic means, and boxes indicate the interquartile range with the median. (**D**) Standardized effects of marker0580 on the four component traits in 2019. Effect sizes are expressed as Cohen’s d for BB relative to AB, with positive values indicating higher trait values in BB individuals. *p* values were calculated using Welch’s *t*-test. Formal marker × year interaction tests for the four traits are reported in Supplementary Table S5.

Genome-wide scans using PC1 as the composite phenotype revealed stronger association signals than those obtained for most individual plant architecture traits. In 2018, the strongest PC1 association was detected at marker0399 on LG8 at 38.237 cM, with a LOD score of 3.06, which remained below the 5% genome-wide permutation threshold of 3.47. In contrast, the 2019 PC1 scan identified a peak at marker0580 on LG3 at 12.516 cM, with a LOD score of 3.67, exceeding the corresponding genome-wide threshold of 3.47 (Figure 5). The marker accounted for approximately 15.5% of the variation in PC1, representing the strongest genome-wide association detected in the present analyses. The BLUE-based PC1 scan also showed its highest peak at marker0580 (LOD = 2.95; approximately 12.7% of variance explained), although this signal did not exceed the BLUE-specific genome-wide threshold of 3.39.

Inspection of the retained downstream genotype coding at marker0580 revealed an unbalanced distribution: the three observed classes labelled BB, AB, and AA contained 80, 19, and 1 individual, respectively. These labels identify the classes in the retained analysis dataset and should not be interpreted as evidence that the mapping population follows a common F_2_ segregation model. To determine whether the significant association was driven by the single rare-class individual, the robustness analysis was repeated after excluding genotype classes represented by fewer than five individuals. Following exclusion of the single AA-labelled individual, the association between marker0580 and the 2019 PC1 remained significant, with a LOD score of 3.24, exceeding the corresponding 5% genome-wide permutation threshold of 3.16. Thus, the 2019 association was not attributable solely to this single rare observation (Figure S2).

We then compared the four component traits between marker0580 genotype classes in 2019. Individuals with the BB genotype showed consistently greater vegetative growth than AB individuals. Mean plant height was 150.35 cm in BB individuals compared with 131.75 cm in AB individuals, representing a difference of 18.60 cm (*p* = 0.00023). Mean tiller length was 89.83 cm and 73.28 cm for BB and AB individuals, respectively, corresponding to a difference of 16.55 cm (*p* = 0.000012). Similarly, tiller diameter increased from 5.83 mm in AB individuals to 6.90 mm in BB individuals (*p* = 0.000039), while mean tiller number increased from 2.84 to 4.27 (*p* = 0.0041). Thus, all four component traits showed concordant genotype effects, consistent with the strong association observed for the composite PC1 phenotype.

The marker0580 signal was substantially weaker in the 2018 dataset, and the genome-wide PC1 peak in that year occurred on LG8 rather than LG3. Formal repeated-measures marker × year tests, conducted after excluding the single AA-labelled genotype, confirmed significant temporal heterogeneity for all four architecture traits: plant height (*p* = 0.00647; FDR-adjusted *p* = 0.00862), tiller length (*p* = 0.00116; FDR-adjusted *p* = 0.00232), tiller diameter (*p* = 0.000228; FDR-adjusted *p* = 0.000912), and tiller number (*p* = 0.0191; FDR-adjusted *p* = 0.0191). Thus, the difference between 2018 and 2019 is supported by an explicit marker × year interaction model rather than by a comparison of nominal significance alone. The LG3 signal was strong and genome-wide significant in 2019 but was not temporally stable across the two seasons (Supplementary Table S5).

### 3.6. Additional Suggestive Associations for Biomass and Physiological Traits

Several weaker associations were detected for biomass-related and physiological traits. None exceeded the corresponding 5% genome-wide permutation thresholds, so these findings were treated as secondary suggestive signals. Marker0907 on LG12 at 57.272 cM was the strongest BLUE-based marker for both single-tiller fresh weight and whole-plant fresh weight (LOD = 2.39 and 1.84; PVE = 10.8% and 8.5%, respectively) and was also the strongest marker for both traits in 2019. However, the signal was not consistently reproduced in 2018 and was partly sensitive to rank-based inverse-normal transformation (Figure S1), indicating substantial year dependence. Because these traits represent fresh rather than dry biomass, short-term differences in plant water status at harvest may also contribute to their variation; the LG12 signal is therefore considered preliminary. For physiological traits, marker0277 on LG3 at 5.114 cM was the strongest BLUE-based marker for both intercellular CO_2_ concentration and intrinsic water-use efficiency (WUEi), with LOD scores of approximately 2.4 and PVE values of 10.6% and 10.7%, respectively. Both traits retained their strongest BLUE-based peak at marker0277 after inverse-normal transformation, but year-specific support was poor; this is therefore interpreted as a shared but suggestive physiological signal rather than independent replication. A further suggestive transpiration-rate peak occurred at marker3163 on distal LG9, but its observed genotype classes were highly unbalanced (AB = 97, BB = 3), so genotype-class effects were not interpreted further. Overall, the biomass and physiological findings were weaker and less temporally consistent than the plant-architecture signals and require validation in larger populations and additional environments (Supplementary Table S4).

## 4. Discussion

### 4.1. Phenotypic Variation and Genetic Complexity of Biomass-Related Traits in the Interspecific Miscanthus Population

The F_1_ population showed broad, mostly continuous variation for agronomic, biomass-related, and physiological traits. Earlier work likewise shows that Miscanthus biomass reflects the combined effects of height, tiller production, stem dimensions, development, and environmental response [13,14,15,26]. None of the single-trait peaks crossed the 5% genome-wide permutation threshold. Several nevertheless explained roughly 8–13% of phenotypic variation and reappeared in related traits or datasets. The simulation-based power analysis provided a quantitative context for these results. The simulated 5% genome-wide threshold was LOD 3.52, close to the empirical thresholds obtained from the phenotype permutations. Under this design, a directly genotyped marker explaining 10% of phenotypic variance was detected in only 23.8% of simulations, whereas power was 53.9% at 15% PVE, 80.7% at 20% PVE, and 94.9% at 25% PVE. Under the simulated conditions, approximately 20% PVE was required to reach 80% power with N = 100 and the present marker set for a directly genotyped causal marker. The full simulated power curve is provided in Figure S3. The observed single-trait peaks with PVE values near 8–13% therefore fall in a range where the simulation indicates limited detection power. This limited power does not provide evidence that sub-threshold peaks are true loci. Moreover, because the simulation assumes a directly genotyped causal marker, actual power may be lower when the assayed marker is only partially linked to an underlying causal variant. Because PVE was estimated from the same small population used for marker detection, these single-marker R^2^ values may also be upwardly biased and should be interpreted as approximate effect-size indicators rather than precise estimates, a limitation that has also been emphasized for small Miscanthus mapping populations [27]. The single-trait results are therefore treated as suggestive signals. Recurrence across traits, years, or alternative phenotype treatments can prioritize regions for follow-up but does not by itself establish a true locus, particularly when the traits are correlated.

### 4.2. A Repeated Suggestive Plant-Architecture Association Cluster on LG9

The proximal part of LG9 contained the most repeatable single-trait pattern. Marker2342 at 11.571 cM showed suggestive signals for plant height and tiller length, and the direction of the genotype-class difference was similar in 2018, 2019, and the across-year adjusted values. Tiller number peaked at the neighboring marker2343 (10.561 cM), although its AA class contained only one individual and was not interpreted further at the genotype-class level. The LOD scores remained below the genome-wide thresholds, so this region is not described as a significant QTL. Formal marker × year tests showed no detectable interaction for marker2342 effects on plant height or tiller length, whereas tiller number showed a significant interaction. The LG9 pattern should therefore be interpreted more narrowly as repeated suggestive evidence for longitudinal vegetative-growth traits rather than as a uniformly stable plant-architecture locus. Because plant height, tiller length, and tiller number are correlated components of plant architecture, recurrence across these traits is not independent replication. Previous mapping studies have likewise reported genetic effects on height and developmental traits that vary in their consistency across years or environments in Miscanthus [15,20,26,28]. At the present marker density, it is also not possible to distinguish pleiotropy from linkage or to determine whether marker2342 and marker2343 tag one underlying locus or separate linked effects. We therefore treat the proximal LG9 interval as a repeated suggestive association region requiring denser mapping and independent validation.

### 4.3. Multivariate Analysis Reveals a Strong but Year-Dependent Plant-Architecture Association on LG3

Marker0580 on LG3 behaved differently. The four architecture traits loaded positively on PC1, which captured 73.20–76.57% of their variation across the 2018, 2019, and BLUE datasets. In 2019, marker0580 at 12.516 cM reached LOD 3.67, above the 5% permutation threshold of 3.47. The result remained significant after the single rare-class individual was removed, and the predominant genotype classes differed consistently across all four component traits. The same pattern was not seen in 2018, and the BLUE peak did not cross the genome-wide threshold. Formal repeated-measures tests confirmed significant marker × year interactions for all four architecture traits after FDR correction. This temporal heterogeneity cannot be interpreted simply as a seasonal environmental response. The 2018 measurements were obtained during the establishment year, whereas 2019 represented an established perennial stand; annual precipitation also differed (388 mm in 2018 versus 443 mm in 2019), with different within-season rainfall distributions [8]. The temporal difference associated with marker0580 may therefore reflect establishment stage, plant age, climatic variation, age-dependent expression, or a combination of these factors. Previous studies have also reported the variation in Miscanthus growth and biomass performance across growing conditions and environments [29,30]. Marker0580 should consequently be viewed as a strong 2019-specific association requiring replication across additional years and environments rather than as a temporally stable selection marker.

### 4.4. Suggestive Genetic Signals for Biomass and Physiological Traits

The biomass and physiological signals were less convincing. Marker0907 on LG12 showed a secondary, year-dependent suggestive signal for single-tiller fresh weight and whole-plant fresh weight, mainly in 2019, but the pattern was weak in 2018 and was partly sensitive to phenotype transformation. Because fresh rather than dry biomass was measured, these traits may also reflect short-term variation in plant water status at harvest. Previous genome-wide association studies have also investigated the genetic basis of biomass yield in diverse Miscanthus germplasm evaluated across multiple environments [31]. Marker0277 on LG3 was the strongest BLUE-based peak for both intercellular CO_2_ concentration and intrinsic water-use efficiency, and its location was retained after inverse-normal transformation, but year-specific support was poor. The previous transcriptomic study of this same cross under semiarid mine-land conditions provides useful context for these physiological responses [9], but it does not validate the marker associations. The distal LG9 transpiration signal at marker3163 was also not interpreted at the genotype-class level because the minor observed class contained only three individuals. These regions are therefore retained only as secondary suggestive findings requiring independent validation.

### 4.5. Implications for Miscanthus Improvement and Study Limitations

From a breeding perspective, statistical strength and repeatability gave different pictures of the two main regions. The proximal LG9 signals were modest, with marker2342 showing no detectable marker × year interaction for plant height and tiller length, whereas marker0580 was stronger in 2019 and showed significant marker × year interactions for all four architecture traits. Neither pattern is ready for marker-assisted use. The study also has clear technical and experimental limits. The population included only 100 F_1_ genotypes, and the 461 mapped SNP positions were unevenly spaced, which limits mapping resolution and the detection of moderate-effect loci; marker density, genotype-calling quality, and marker order are important determinants of mapping precision in outcrossing Miscanthus populations [32]. Although the retained genotype matrix allows observed genotype-class configurations to be reported, the original parental phase assignments and complete upstream filtering records are unavailable, precluding retrospective reconstruction of a fully phased interval-mapping analysis [33]. The results are therefore reported as mapped marker–trait associations rather than conventional interval-mapped QTLs. In addition, 2018 was the establishment year and 2019 represented an established perennial stand, so temporal differences cannot be attributed exclusively to seasonal climatic variation. Residual spatial heterogeneity within the reclaimed field also cannot be excluded because no formal pre-plant spatial survey or fine-scale spatial covariates were available. All genotypes were evaluated under the same reclaimed mine-land condition, and no contrasting agricultural or non-reclaimed site was included; the detected associations therefore represent genetic variation expressed at this site rather than loci demonstrated to confer mine-land-specific adaptation. Future validation should use larger populations, denser markers, additional years and contrasting environments, and would also benefit from repeated, standardized high-throughput phenotyping of dynamic plant architecture.

## 5. Conclusions

This two-year field study identifies a small set of genomic regions for follow-up in an interspecific Miscanthus F_1_ population evaluated at a reclaimed mine-land site. No single-trait peak reached the genome-wide permutation threshold. The proximal LG9 interval showed repeated suggestive associations with plant-architecture traits, involving marker2342 for plant height and tiller length and the neighboring marker2343 for tiller number; none of these single-trait signals exceeded the corresponding genome-wide threshold. By contrast, marker0580 on LG3 reached genome-wide significance for architecture PC1 in 2019 and remained significant after exclusion of the singleton AA-labelled class, although formal marker × year tests demonstrated temporal heterogeneity. Biomass and physiological associations were weaker and less repeatable. Because the first season represented stand establishment, all genotypes were tested at only one reclaimed site, and the linkage map is relatively sparse, these findings should be interpreted as marker–trait relationships expressed under the conditions of this experiment rather than as validated loci for mine-land adaptation or marker-assisted selection. Validation in larger populations with denser marker coverage, additional years, and contrasting environments is needed before the LG9 and LG3 regions can be resolved biologically or evaluated for breeding use.

## Figures and Tables

**Table 1:** Descriptive statistics of agronomic, biomass-related, and physiological traits in the interspecific Miscanthus F_1_ population.

Trait	N	Mean	SD	Min	Max	CV (%)	Skewness	Kurtosis	Shapiro–Wilk P
Single_tiller_fresh_weight	96	9.09	4.01	2.42	20.77	44.17	0.77	2.97	1.60 × 10^−4^
Whole_plant_fresh_weight	96	22.80	17.00	3.31	89.07	74.60	1.63	5.62	5.70 × 10^−9^
Plant_height	100	131.55	17.04	86.00	168.50	12.95	−0.13	2.62	0.709
Tiller_length	100	74.52	14.32	40.94	105.87	19.21	0.10	2.56	0.431
Tiller_diameter	100	6.15	0.97	3.37	8.37	15.71	−0.06	3.60	0.072
Tiller_number	100	3.44	1.84	1.00	9.00	53.29	1.18	3.77	2.78 × 10^−7^
Leaf_length	100	12.95	2.42	9.25	32.00	18.72	4.90	41.30	2.64 × 10^−14^
Leaf_width	100	1.29	0.17	0.90	1.68	13.14	0.05	2.48	0.712
Leaf_area	100	52.03	9.81	28.97	91.63	18.85	0.56	4.43	8.874 × 10^−3^
Leaf_angle	100	17.83	4.46	9.00	29.00	25.01	0.34	2.73	0.179
Photosynthetic_rate	100	18.90	2.63	12.24	27.43	13.93	0.20	4.00	0.2257
Stomatal_conductance	100	0.13	0.02	0.08	0.19	15.42	−0.13	3.37	0.2324
Intercellular_CO_2_	100	125.57	21.71	13.52	179.17	17.29	−1.79	11.37	5.56 × 10^−8^
Transpiration_rate	100	3.40	0.69	1.78	5.33	20.28	−0.11	3.03	0.121
WUEe	100	5.95	1.07	4.49	10.86	17.96	1.86	8.02	6.66 × 10^−9^
WUEi	100	150.56	13.36	117.29	219.96	8.87	2.05	12.57	6.75 × 10^−9^

Note: For traits measured in both 2018 and 2019, descriptive statistics were calculated from the mean phenotype of each F_1_ genotype across the two growing seasons. Annual genotype values were arithmetic means of three measured plants. Single-tiller fresh weight and whole-plant fresh weight had N = 96 because four genotypes lacked measurements in both years; the remaining two-year traits had N = 100. Leaf angle was measured only in 2019 (N = 100). CV, coefficient of variation; WUEe, instantaneous water-use efficiency (A/E); WUEi, intrinsic water-use efficiency (A/g_s_). Kurtosis is reported as Pearson kurtosis, with a normal distribution having a kurtosis of 3.

**Table 2:** Summary statistics of the genetic linkage map used for genome-wide marker–trait analysis in the interspecific Miscanthus F_1_ population.

Linkage Group	No. of Markers	Length (cM)	Average Interval (cM)	Largest Interval (cM)
1	43	206.31	4.91	25.54
2	29	228.63	8.17	38.83
3	16	95.03	6.34	23.90
4	26	226.65	9.07	43.38
5	12	115.18	10.47	41.05
6	19	165.64	9.20	34.66
7	43	217.23	5.17	22.32
8	21	138.08	6.90	27.24
9	48	231.95	4.94	32.70
10	17	188.80	11.80	48.38
11	32	140.59	4.54	13.72
12	25	207.33	8.64	34.66
13	14	110.39	8.49	36.70
14	12	171.73	15.61	91.63
15	26	140.61	5.62	22.31
16	21	139.24	6.96	34.66
17	19	158.38	8.80	30.81
18	22	140.34	6.68	20.78
19	16	193.47	12.90	38.83
Total	461	3215.56	7.27	91.63

**Table 3:** Major suggestive marker–trait association peaks identified from genome-wide scans of agronomic, biomass-related, and physiological traits in the interspecific Miscanthus F_1_ population.

Trait	Dataset	LG	Peak Marker	Position (cM)	LOD	PVE (%)	Trait Category
Plant height	BLUE	9	marker2342	11.571	2.26	9.9	Plant architecture
Tiller length	BLUE	9	marker2342	11.571	2.51	10.9	Plant architecture
Tiller number	BLUE	9	marker2343	10.561	2.13	9.4	Plant architecture
Single-tiller fresh weight	BLUE	12	marker0907	57.272	2.39	10.8	Biomass
Whole-plant fresh weight	BLUE	12	marker0907	57.272	1.84	8.5	Biomass
Intercellular CO_2_	BLUE	3	marker0277	5.114	2.43	10.6	Physiology
WUEi	BLUE	3	marker0277	5.114	2.45	10.7	Physiology
Transpiration rate	BLUE	9	marker3163	168.900	2.46	10.7	Physiology

None of the individual-trait association peaks exceeded the trait-specific 5% genome-wide significance threshold determined by 1000 permutations. The regions listed here are therefore treated as suggestive rather than significant QTLs.

## Data Availability

The phenotype, retained marker-genotype data, linkage-map positions, and analysis outputs supporting the findings of this study are available within the article, its Supplementary Materials, or from the corresponding authors upon reasonable request. Historical intermediate files from the original linkage-map construction are not part of the retained analysis dataset used for the present reanalysis.

## Supplementary Materials

The following supporting information can be downloaded at: https://www.mdpi.com/article/10.3390/plants15203172/s1, Figure S1, sensitivity analysis after rank-based inverse normal transformation; Figure S2, robustness analysis of the marker0580 association after exclusion of the rare genotype class; Figure S3, simulation-based power analysis; Table S1, mapped SNP markers and genetic positions; Table S2, year-specific descriptive statistics and cross-year correlations; Table S3, leading marker–trait peaks from annual and across-year scans; Table S4, multi-trait genomic regions; and Table S5, plant-architecture PC1 loadings, formal marker × year interaction tests, observed genotype-class configurations across the retained marker set, and genotype-class counts for principal markers.
