# Supplementary material for: Genome-Wide Marker–Trait Analysis of Agronomic and Biomass-Related Traits in an Interspecific Miscanthus F1 Population Grown on Reclaimed Mine Land
Source: Plants (Basel). 2026 Oct 16;15(20):3172. doi: 10.3390/plants15203172 (PMC13709115; doi:10.3390/plants15203172)
Supplement: Supplementary file 1 [file plants-15-03172-s001.zip › plants-4595274-supplementary.pdf]

Sensitivity of genome-wide marker–trait scans to rank-based inverse normal transformation

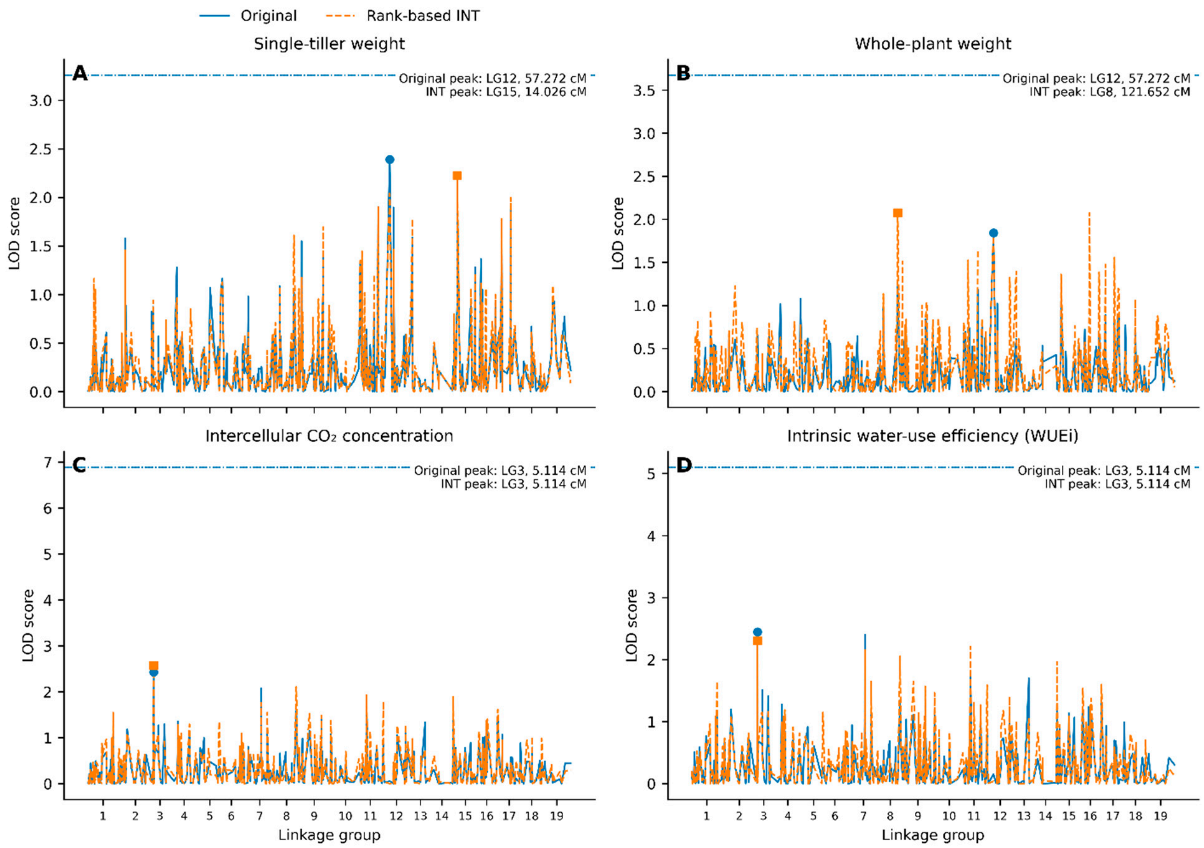

Figure S1. Sensitivity analysis of genome-wide marker–trait associations following rank-based inverse normal transformation. Genome-wide LOD profiles based on the original BLUE phenotypic values and rank-based inverse normal transformed (INT) values are compared for (A) single-tiller weight, (B) whole-plant weight, (C) intercellular CO_2 concentration, and (D) intrinsic water-use efficiency (WUEi). Horizontal reference lines indicate the corresponding 5% genome-wide permutation thresholds. The locations of the strongest association peaks from the original and transformed analyses are indicated in each panel.

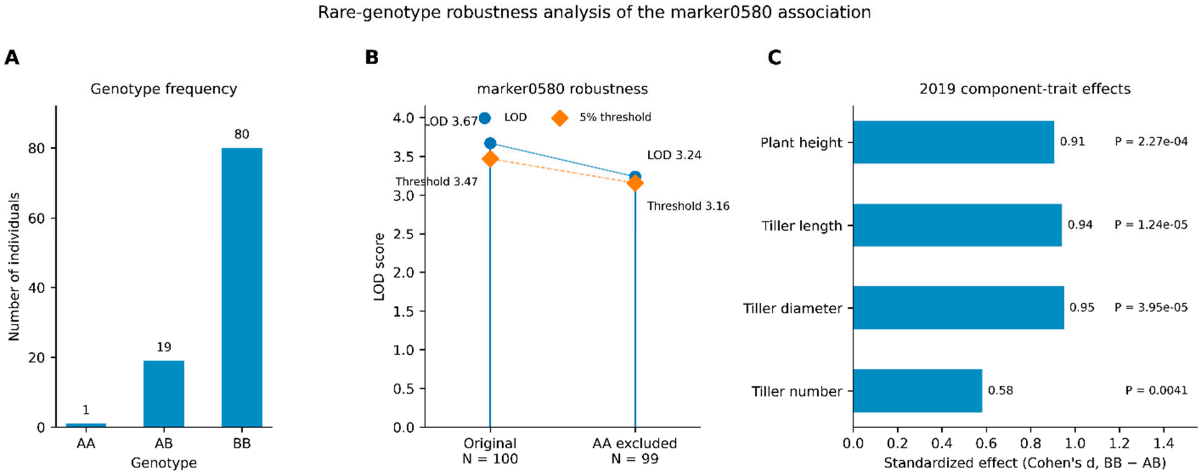

Figure S2. Robustness analysis of the marker0580 association with plant architecture in 2019. (A) Genotype frequencies at marker0580 in the F_1 population. The AA genotype was represented by only one individual, whereas AB and BB were represented by 19 and 80 individuals, respectively. (B) Comparison of the original genome-wide association result with the analysis after exclusion of the rare AA genotype. The marker0580 association remained above the corresponding 5% genome-wide permutation threshold after exclusion of the rare genotype class (LOD = 3.24; threshold = 3.16; n = 99). (C) Standardized genotype effects (Cohen's d, BB − AB) for the four component traits of plant architecture in 2019. Positive values indicate higher trait values in the BB genotype class.

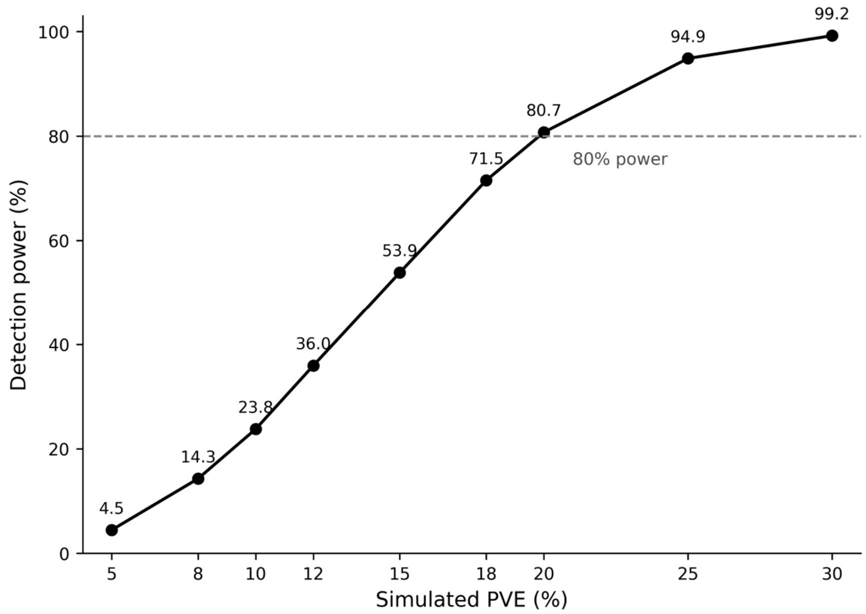

Figure S3. Simulation-based power analysis for genome-wide marker–trait detection in the F_1 population. Detection power was estimated using the observed genotype structure of the mapping population and the categorical single-marker model. Ten thousand null simulations were used to estimate the 5% genome-wide maximum-LOD threshold (LOD ≈ 3.52), and 5,000 simulations were conducted at each assumed true marker effect (PVE = 5–30%). Power was defined as the proportion of simulations in which the causal marker exceeded the simulated genome-wide threshold. Detection power increased from 23.8% at 10% PVE to 53.9% at 15% PVE and 80.7% at 20% PVE, indicating that, under the simulated conditions, a directly genotyped causal marker needed to explain approximately 20% of phenotypic variance to achieve 80% power.
